# Dysregulation of miR‐543 in Parkinson's disease: Impact on the neuroprotective gene SIRT1

**DOI:** 10.1111/nan.12864

**Published:** 2022-11-26

**Authors:** Mirte Scheper, Anand Iyer, Jasper J. Anink, Lucia Mesarosova, James D. Mills, Eleonora Aronica

**Affiliations:** ^1^ Department of (Neuro)Pathology Amsterdam Neuroscience Amsterdam UMC Location University of Amsterdam Amsterdam The Netherlands; ^2^ Department of Internal Medicine Erasmus MC Rotterdam The Netherlands

**Keywords:** differential expression, grey matter, microRNAs, Parkinson's disease, SIRT1, white matter

## Abstract

**Aims:**

Parkinson's disease (PD) is a progressive and age‐dependent neurodegenerative disease characterised clinically by a variety of motor symptoms and cognitive impairment. PD was initially considered to be a grey matter disease; however, recently, evidence has emerged that white matter changes in PD precede the neuronal loss seen in the grey matter. The cause of these initial white matter changes is yet to be elucidated. Here, we explored whether dysregulated miRNAs and their target mRNA could provide insight into the underlying mechanisms of early white matter changes in PD.

**Methods:**

We analysed the expression of miRNAs in three different stages of PD through RNA‐sequencing and validated the differential expression of miRNAs through quantitative reverse transcription polymerase chain reaction. With bioinformatic analyses, we predicted target genes of dysregulated miRNAs and investigated their biomarker potential. Finally, in vitro, we confirmed the targetting of the gene *SIRT1* by miR‐543.

**Results:**

We identified 12 dysregulated miRNAs in PD and found that miR‐543 holds potential as a biomarker for late‐stage PD with dementia. We report upregulation of miR‐543 in early PD white matter tissue and downregulation of *SIRT1*. In vitro experiments showed that the upregulation of miR‐543 results in the downregulation of SIRT1 in the white matter, but not in the grey matter.

**Conclusions:**

We validated *SIRT1* as a target of miR‐543 in the brain and showed its function as a potential biomarker. Our results highlight the idea that dysregulation of miR‐543 in early PD white matter, resulting in the dysregulation of SIRT1, potentially influencing the early white matter changes observed in PD.

## Graphical abstract

In this study we explored miRNA dysregulation in Parkinson's disease (PD) and identified the dysregulation of miR‐543 in the white matter. Overexpression of miR‐543 in the white matter of individuals with PD may lead to a downregulation of the neuroprotective gene *SIRT1*, possibly contributing to the early white matter changes observed in PD. Here, we also show that miR‐543 may potentially have utility as a biomarker for identifying individuals with PD and dementia.

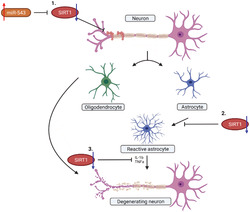

**Key Points**

- *SIRT1* is a target of miR‐543 in the brain, predominantly in the white matter.
- Overexpression of miR‐543 in the white matter and subsequent downregulation of SIRT1 possibly impacts early observed white matter changes in PD.
- miR‐543 holds potential as a biomarker for late‐stage PD with dementia.

## INTRODUCTION

Parkinson's disease (PD) is a progressive and age‐dependent neurodegenerative disease [1]. PD is a disease that steadily increases in incidence as individuals age. Among women aged 60 to 69, this incidence is 30 out of 100,000 and for men, it stands at 58 out of 100,000. For those over 80, the incidence among women is 80 out of 100,000 and for men, 258 out of 100,000 [2]. PD manifests with both motor and non‐motor symptoms, with the former being the most common. The motor symptoms include resting tremor, bradykinesia, postural instability and rigidity [3]. Furthermore, non‐motor symptoms have become more acknowledged over time as they can be similarly debilitating as the motor symptoms. Non‐motor symptoms in PD include cognitive decline, depression, anxiety, dysautonomia and sleep disturbances [4]. With this broad spectrum of symptoms, PD has wide‐ranging social and economic impacts resulting in a decrease in the quality of life of patients and their surroundings.

The main neuropathological hallmarks of PD include increasing accumulation and aggregation of alpha‐synuclein (α‐Syn) protein and the loss of the nigrostriatal dopaminergic neurons [5]. The resulting motor abnormalities mark the progression of PD, whereas non‐motor symptoms can already be identified in the prodromal stages of the disease. Due to this characterisation, PD was originally classified as a grey matter disorder. However, recent studies have introduced the idea that white matter changes occur prior to the degenerative loss of neuronal cell bodies [6]. Axon degeneration and alterations in axonal transport are considered to be the earliest and most predominant features of PD [7]. Further evidence of early white matter changes comes from imaging studies implying that white matter integrity is affected in PD patients. These studies have shown that microstructural changes in the white matter could reflect the loss of white matter because of demyelination and axonal damage [8, 9]. The cause of these initial white matter changes is yet to be elucidated; however, initial axonal damage may be attributed to the synaptic accumulation of toxic α‐Syn.

Recently, there has been an increasing number of studies showing that microRNAs (miRNAs) play an important role in post‐transcriptional regulation of genes that are associated with the pathogenesis of several diseases [10]. miRNAs are small non‐coding RNAs that interact with 3′ untranslated regions (UTRs), 5′ UTRs, gene promoter regions and coding sequences of their target mRNAs, resulting in degradation and regulation of transcription and translation [11, 12, 13]. In PD, multiple miRNAs are differentially expressed compared to healthy controls. Dysregulated miRNAs in PD are related to the regulation of PD‐associated genes such as synuclein alpha (*SNCA*), parkin RBR E3 ubiquitin protein ligase (*PRKN*), PTEN Induced Kinase 1 (*PINK1*) and more. Furthermore, miRNAs related to neuroinflammation and dopaminergic neuron survival have also been found to be dysregulated in PD [14]. Hence, disturbance of miRNA expression could potentially contribute to the pathogenesis of PD through modulation of PD‐associated gene and protein expression. Previous studies have identified several differentially expressed miRNAs in PD brain tissue; however, their role in disease pathogenesis has not been fully elucidated [15, 16].

Thus, we hypothesise that dysregulated miRNAs may be involved in the establishment and progression of PD through the targeting and regulation of PD‐associated genes. Therefore, this study set out to determine differentially expressed miRNAs in the middle frontal gyrus (MFG) of PD patients. Tissue was selected from the MFG as it is affected in the later stages of PD, predominately in Braak stages 5 and 6, allowing the determination of the molecular events prior to the serious cellular loss in PD pathology. Next, we looked to identify and functionally characterise promising miRNA targets. Moreover, we also assessed the feasibility of using miRNAs as cerebral spinal fluid (CSF)‐based biomarkers for PD.

## METHODS

### Cohort

The middle frontal gyrus was selected and obtained from the Netherlands Brain Bank (NBB), Netherlands Institute for Neuroscience, Amsterdam. The material was collected from donors with written informed consent for the use of the material and clinical information for research purposes. For all samples (Table 1), brain tissue was either frozen, separated into grey and white matter using the cryostat and kept at −80° (for RNA isolation) or fixed in 4% paraformaldehyde and embedded in paraffin (FFPE, for staining experiments). Moreover, the CSF of patients was obtained from the same patients. All samples and the experiments they used are listed in supporting information Table S1. Tissue was obtained and used in accordance with the Declaration of Helsinki and the Amsterdam UMC Research Code provided by the Medical Ethics Committee, and the study was approved by the local ethical committees of all participating medical centres.

**TABLE 1:** Demographic and details of PD patients and non‐demented controls

Variables (*N*)	Controls	PD4	PD5/6	PDD5/6
Number of samples	19	16	9	19
Age, (mean ± SD), y	89.43 ± 11.22	71 ± 11.75	73.3 ± 9.15	78.8 ± 6.01
Male, *n* (%)	9 (47%)	8 (50%)	6 (60%)	13 (65%)
Tissue pH	6.59	6.63	6.44	6.38
Post‐mortem delay (h:min)	06:20	06:21	05:05	05:55
Brain weight (g)	1204	1227	1291	1249

*Note*: Donors were classified based on their final clinical diagnosis as PD [17]. The autopsy was performed using a standardised protocol by the NBB (open access: www.brainbank.nl and the presence of neuropathological features were assessed following consensus criteria for diagnosis of PD [18, 19]. PD, Parkinson's disease; NBB, Netherlands Brain Bank.

### RNA‐sequencing

Cortical specimens from non‐demented controls and PD patients were snap‐frozen in liquid nitrogen and stored at −80°C until use for RNA isolation. Tissue for RNA‐sequencing (RNA‐Seq) was divided into three groups: non‐demented controls (WM: *n* = 9, GM: *n* = 10), Braak 4 (WM: *n* = 6, GM: *n* = 10) and Braak 5/6 (WM: *n* = 9, GM: *n* = 9). Frozen tissue material (separated in grey and white matter) was homogenised in Qiazol Lysis Reagent (Qiagen Benelux, Venlo, The Netherlands). The total RNA including the miRNA fraction was isolated using the miRNeasy Mini kit (Qiagen Benelux, Venlo, the Netherlands) according to the manufacturer's instructions. The concentration and purity of RNA were determined at 260/280 nm using a Nanodrop 2000 spectrophotometer (Thermo Scientific, Wilmington, DE, USA), and RNA integrity was assessed using a Bioanalyser 2100 (Agilent). Samples required a RNA integrity number (RIN) greater than 6.0 for use in downstream sequencing. All library preparations and sequencing were completed at GenomeScan (Leiden, the Netherlands). Samples were processed for small RNA‐Seq using the TruSeq Small RNA‐Seq preparation kit (Illumina, San Diego, CA, USA) in accordance with manufacturers' guidelines. In brief, small RNA was isolated from purified RNA by size selection after the ligation of sequencing adapters. After gel excision, the selected RNA fragments were amplified by PCR. All clustering and DNA sequencing used the Illumina cBot and the HiSeq 4000. All samples sent for small RNA‐Seq were subjected to paired‐end sequencing with a read length of 151 nucleotides to a depth of 12 million reads per sample.

### Read quality and alignment

Read quality was assessed using FastQC version 0.11.8 software produced by the Babraham Institute (Babraham, Cambridgeshire, UK), and Trimmomatic v0.36 was used to filter low‐quality base calls and any adapter contamination [20] Low‐quality leading and trailing bases were removed from each read, a sliding window trimming using a window of four and a phred33 score threshold of 15 was used to assess the quality of the read body. Any reads of < 17 nucleotides were discarded.

Reads were aligned to the human reference genome, GRCh38 using Bowtie2 version 2.2.6 [21]; no mismatches between the seed sequence and the reference genome were allowed, and reads were allowed to align a maximum of 10 times. Using the featureCounts programme from the Subread package version 1.6.4, the number of reads that aligned to the miRNAs, according to miRBase22 [22, 23] and other short RNA species extracted from Gencode v31 were calculated [24, 25]. miRNAs with ≥ 1 read count in at least one of the samples were kept for analysis. Differential expression analysis was performed using the R package DESeq2 [26]. As RNA‐Seq was performed in two batches, the batch was included in the DESeq2 design formula, this allow for the statistical inferences to be adjusted for the batch. The false discovery rate was controlled for using the Benjamini–Hochberg correction, and gene expression changes with an adjusted *p*‐value < 0.05 were considered statistically significant.

### miRNA target prediction

Potential targets of differentially expressed miRNAs were explored using the target gene prediction algorithm TargetScan [27]. The correlation between the expression of the miRNA and the expression of the predicted target genes thought to bind in the 3′ UTR was determined. Target genes that were negatively correlated (<0.6) with their respective miRNA were used for further analysis. To further narrow down the list of potential target genes, pathway enrichment analysis was performed. miRNA regulatory target gene enrichment analysis was performed through gene ontology (GO) and Kyoto Encyclopedia of Genes and Genomes (KEGG) [28], with a cut‐off criterion of adjusted *p*‐value < 0.05.

### Assessment of biomarker usage

To determine the potential biomarker properties of miRNAs, the expression profile of chosen miRNAs was used for a Receiver Operation Characteristic (ROC) curve analysis. This method was used to display the discriminatory accuracy of the miRNA to distinguish between controls and PD with an Area Under the Curve (AUC). The analyses were carried out using the ‘pROC’ package in R studio [29].

To assess the robustness of miR‐543 as a classifier, a permutation analysis was performed. For each permutation, the samples were randomly assigned to groups, and an ROC analysis was performed for miR‐543. The AUC for each permutation was recorded, and this was repeated 30,000 times to produce a test statistic distribution. Finally, by observing where the original AUC fell within this distribution, the *p*‐value was calculated.

### RNA isolation and quantitative real‐time PCR

For the validation of miRNA expression from RNA‐Seq, an independent cohort was selected and divided into four groups: non‐demented controls (*n* = 9), PD patients in Braak stage 4 (PD4) (*n* = 6), PD patients in Braak stage 5/6 (PD5/6) (*n* = 9) and PD patients with dementia in Braak stage 5/6 (PDD5/6) (*n* = 10).

For RNA isolation from tissue, 700 μl Qiazol Lysis Reagent (Qiagen Benelux, Venlo, The Netherlands) was used to homogenise each sample. Total RNA was isolated using the miRNeasy Mini kit (Qiagen Benelux, Venlo, The Netherlands) according to the manufacturer's instructions. The concentration of the RNA was determined at 260/280 nm using the NanoDrop 1000 Spectrophotometer (Thermo Fisher Scientific, Wilmington, DE, USA). For the evaluation of mRNA expression, 250 ng of total RNA was reverse‐transcribed into cDNA using oligo‐dT primers, and for the evaluation of miRNA expression, 100 ng of total RNA was used to generate cDNA using TaqMan MicroRNA reverse transcription kit (Applied Biosystems, Foster City, CA, USA) according to the manufacturer's instructions. TaqMan quantitative reverse transcription polymerase chain reactions (RT‐qPCRs) were run on a Roche Lightcycler 480 thermocycler (Roche Applied Science, Basel, Switzerland) using small nuclear RNA (*RNU6B*) for miRNAs as a reference gene. For other genes, qPCRs were run on a Roche Lightcycler 480 thermocycler (Roche Applied Science, Basel, Switzerland) using elongation factor 1‐α (*EF1‐ α*) as a housekeeping gene for mRNA analysis. The samples (in triplicates) were amplified according to protocol. For RNA isolation from CSF, RNA was isolated as described above, and TaqMan RT‐qPCRs were run with cel‐miR‐39 as a reference gene for analysis.

For RNA isolation from cultures, RNA was isolated as described above. For the evaluation of mRNA expression, qPCRs were run with *EF1‐a* as a housekeeping gene and for the evaluation of miRNA expression, TaqMan RT‐qPCRs were run using *RNU6B* as a reference gene for analysis.

Quantification of data was performed using the computer programme LinRegPCR in which linear regression on the Log (fluorescence) per cycle number data is applied to determine the amplification efficiency per sample [30]. With the use of triplicates for each sample, the average of the *N*_0_ values was taken in the target gene and the reference gene. The *N*_0_ ratio is the ratio between the averages of the target and reference gene for the same sample. For the relative expression, all groups were compared to the controls.

### Immunohistochemistry

Human brain tissue fixed in 10% buffered formalin and embedded in paraffin was mounted on pre‐coated glass slides (Star Frost, Waldemar Knittel, Braunschweig, Germany). Sections were deparaffinised in xylene, rinsed in ethanol (100%, 100%, 96%) and incubated for 20 min in 0.3% hydrogen peroxide diluted in methanol to block endogenous peroxidase activity. Antigen retrieval was performed using a pressure cooker in 0.01 M sodium citrate buffer (pH 6.0) at 120°C for 10 min. Slides were cooled in ice water for 15 min, washed with phosphate‐buffered saline (PBS, pH 7.4) and incubated overnight with primary antibodies against SIRT1 (monoclonal mouse, Abcam, ab110304, 1:200) in antibody diluent at 4°C. Sections were washed in PBS and then stained with a polymer‐based peroxidase immunohistochemistry detection kit (Brightvision plus kit, ImmunoLogic, Duiven, the Netherlands) according to the manufacturer's instructions. Bright 3,3′‐diaminobenzidine + (DAB+) substrate solution was used to perform staining. Distilled water was used to stop the reactions. Sections were counterstained with Haematoxylin–Mayer solution (Klinipath, Breda, the Netherlands), dehydrated in alcohol and xylene and coverslipped. Antibodies used can be found in supporting information Table S4.

### Pathological evaluation of immunostaining

All labelled tissue sections were semi‐quantitatively and quantitatively evaluated. The intensity of the staining was evaluated using a scale between 0 and 3 (0: no; 1: weak; 2: moderate; 3: strong staining) as described previously [31]. For all samples, multiple areas were used to confirm the predominant cell staining intensity. Next, the frequency of SIRT1 positive cells was determined (0: 0%, 1: <10%, 2: 10–40%, 3: 40–80%, 4: >80%). The overall immunoreactivity score (IRS) was calculated by multiplying the intensity score by the frequency score. For quantitative analysis, QuPath software [32] for Windows was used. Images taken (magnification: 20X) with a microscope were loaded into QuPath, and the image type was set to Brightfield (HDAB) for all images. In the annotation menu, the entire image was selected for analysis. Through the analyse menu, positive cell detection was used to determine the percentage of positive cells in each image. The intensity threshold was set to 0.1, with a maximum background intensity of 2. Further adjusted intensity threshold parameters were threshold 1+: 0.05, threshold 2+: 0.1 and threshold 3+: 0.2. All other parameters were standard. Three to five images were analysed for each sample, and the average positive percentage was taken as the final value.

### Cell cultures and transfection

SH‐SY5Y neuroblastoma cells were cultured in Dulbecco's modified Eagle's medium (DMEM)/HAM F12 (1:1) (Gibco, Life Technologies, Grand Island, NY, USA) supplemented with 50 units/ml penicillin, 50 μg/ml streptomycin, 1% L‐Glutamine and 10% foetal calf serum (FCS; Gibco, Life Technologies, Grand Island, NY, USA). Foetal astrocytes were cultured in DMEM/F10 (1:1) (Gibco, Life Technologies, Grand Island, NY, USA) supplemented with 50 units/ml penicillin, 50 μg/ml streptomycin, 1% L‐Glutamine and 10% foetal calf serum (FCS; Gibco, Life Technologies, Grand Island, NY, USA). All cultures were grown and maintained in a 5% CO_2_ incubator at 37°C. For experiments, cells were seeded in 6‐well plates with 5 × 10^4^ cells/well and allowed to adhere for 72 h. After 72 h, cells were transfected with mirVana® miRNA mimic, hsa‐miR‐543 (Thermo Fisher Scientific, Wilmington, DE, USA). Oligonucleotides were delivered to the cells using Lipofectamine® 2000 transfection reagent (Life Technologies) in a final concentration of 50 nM for a total of 24 h for both mRNA and protein experiments. The viability of the cells was checked under the microscope, and cells were harvested 24 h after transfection for mRNA and 48 h for protein determination to ensure an appropriate time for protein turnover. Data of miR‐543 transfected cells were normalised to the lipofectamine control group. This control group consisted of cells exposed to lipofectamine without the presence of oligonucleotides. Data are expressed as a fold‐change compared to the control group.

### Western blot

For protein analysis, cell cultures were lysed using radioimmunoprecipitation assay (RIPA) buffer supplemented with protease inhibitors. Homogenates were centrifuged at 13,000 *g* for 10 min at 4°C and supernatant was used for further analysis. Protein concentrations were determined using the bicinchoninic acid assay (BCA) (Thermo Fisher Scientific, Wilmington, DE, USA). For western blotting, samples were prepared for 10 min at 70°C with 4X lithium dodecyl sulphate (LDS), 1 M dithiothreitol (DTT) and H_2_O. Equal amounts of protein lysate (20 ug/lane) were loaded onto sodium dodecyl sulphate‐polyacrylamide gel electrophoresis on a gradient Bolt 4–12% Bis‐Tris gel (Thermo Fisher Scientific, Waltham, MA, USA) and ran on 100 V for 90 min and transferred to polyvinylidene difluoride (PVDF) membranes (Immobilon‐P; Merck, Darmstadt, Germany) for 60 min at 100 V. Blots were then blocked with 5% bovine serum albumin (BSA) in Tris‐buffered saline with 0.1% Tween20 (TBS‐T; 20 nM Tris, 150 mM NaCl, 0.1% Tween20, pH 7.5) for 1 h at RT. Membranes were then incubated with primary antibodies against SIRT1 (monoclonal mouse, Abcam, ab110304, 1:1000) overnight at 4°C in 5% BSA in TBS‐T and subsequently washed 3 × 10 min in TBS‐T. GAPDH was used as a loading control (1:2000, Sigma–Aldrich, A3854). Next, membranes were incubated with horseradish peroxidase‐coupled secondary antibodies for 1 h at RT in TBS‐T (P044701 and P04480, 1:10000, DAKO). After three washes in TBS‐T, membranes containing GAPDH were incubated with ECL western blotting detection reagent (Thermo Fisher Scientific, Wilmington, DE, USA), and membranes containing SIRT1 were incubated with ECL PLUS western blotting detection reagent (Thermo Fisher Scientific, Wilmington, DE, USA). Blots were digitised using the ImageQuant LAS 4000 system (GE Healthcare Europe, Eindhoven, The Netherlands). The Gel Analyser tool of ImageJ was used to determine the profiles of each lane of the gels, and values of SIRT1 were normalised using the values of GAPDH [33].

### Statistical analysis

GraphPad Prism software version 5.01 (GraphPad Software Inc., La Jolla, CA, USA) was used for statistical analysis. Analysis of expression validation was performed using a one‐way ANOVA with a post hoc Tukey honest significance test (HSD) or the non‐parametric Kruskal–Wallis test with correction for multiple comparisons (Dunn's method). A *p*‐value < 0.05 indicated a statistically significant difference. Data for expression validation are shown as box plots for grey and white matter with mean ± SEM.

## RESULTS

### Differential expression and validation of miRNAs in PD

A total of 2303 miRNAs were identified as expressed in the MFG of controls and PD patients. To determine differentially expressed miRNAs in PD patients, differential expression (DE) analysis was performed. After batch correction and normalisation, a total of 12 differentially expressed miRNAs were identified in PD patient samples. These miRNAs consisted of nine downregulated and three upregulated miRNAs (supporting information Figure S1). Cell‐type composition can function as a potential confounding factor when analysing expression patterns. Therefore, we also assessed the cohort for the expression of specific cell‐type markers (supporting information Figure S2). No differences were found in the expression of these markers, indicating that the results found are not because of the cell‐type composition of the samples. For further investigation, three miRNAs with the highest levels of dysregulation were chosen, consisting of one upregulated and two downregulated miRNAs.

**FIGURE 1:**
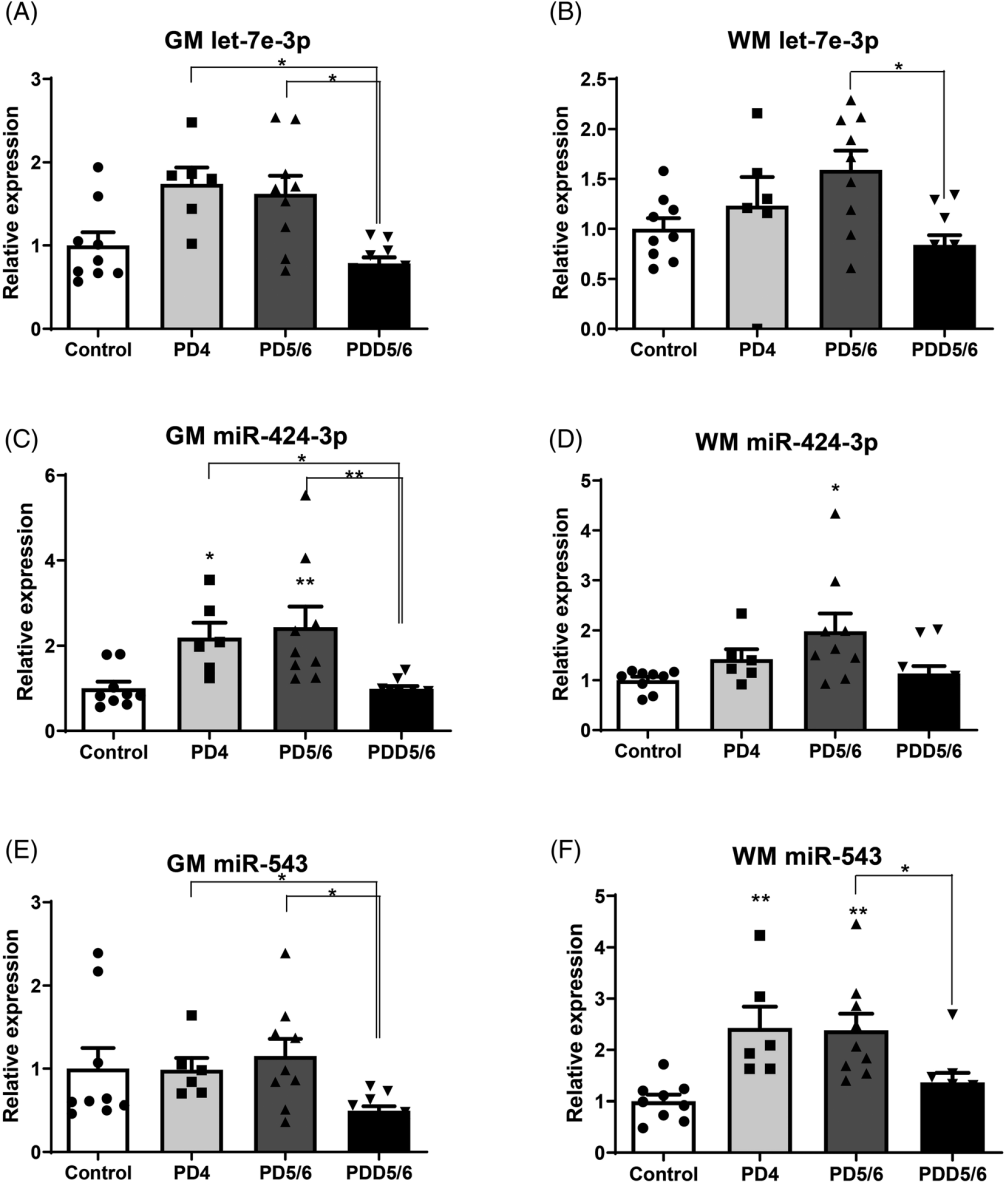
miRNA expression of let‐7e‐3p, miR‐424‐3p and miR‐543 in grey (GM) and white matter (WM) across control and PD groups. (A) Relative expression of let‐7e‐3p in the grey matter across control and PD groups. (B) Relative expression of let‐7e‐3p in the white matter across control and PD groups. (C) Relative expression of miR‐424‐3p in the grey matter across control and PD groups. (D) Relative expression of miR‐424‐3p in the white matter across control and PD groups. (E) Relative expression of miR‐543 in the grey matter across control and PD groups. (F) Relative expression of miR‐543 in the white matter across control and PD groups. *X* axis: relative expression compared to controls. *Y* axis: sample groups. * = *p* < 0.05, ** = *p* < 0.01. Control: *n* = 9, PD4: *n* = 6, PD5/6: *n* = 9, PDD5/6: *n* = 10.

Next, we compared miRNA expression profiles for let‐7e‐3p, miR‐424‐3p and miR‐543 in three different PD stages with control samples. The expression of these three miRNAs was validated using RT‐qPCR on an independent cohort. A significant upregulation of let‐7e‐3p expression was identified in the grey matter in PD4 and PD5/6 compared to the PDD5/6, and similar results were found in the white matter when comparing the PD5/6 and PDD5/6 group (Figure 1A and B). For miR‐424‐3p, upregulation was determined in PD4 in the grey matter, and in PD5/6 upregulation was found in both grey and white matter compared to the controls. Moreover, in the grey matter, PD4 and PD5/6 showed significant upregulation compared to the PDD5/6 group (Figures 1C and D). Finally, miR‐543 was only found to be upregulated in the white matter in PD4 and PD5/6, whereas no significant differences were found in the grey matter when comparing PD groups to the controls (Figure 1E and F). This finding possibly implicates the dysregulation of miR‐543 in the early white matter changes observed in PD patients; this is in contrast with the two other differentially expressed miRNAs, which do not show dysregulation in the white matter in the PD4 group.

### Biomarker potential of let‐7e‐3p, miR‐424‐3p and miR‐543 in CSF

RT‐qPCR was used to determine the expression of let‐7e‐3p, miR‐424‐3p and miR‐543 in CSF of controls (*n* = 9) and PD patients (PD4: *n* = 6, PD5/6: *n* = 9, PDD5/6: *n* = 10). For miR‐424‐3p, the miRNA was below detection levels in CSF and was therefore excluded from further biomarker analysis. In order to evaluate the diagnostic accuracy of let‐7e‐3p and miR‐543, we carried out a ROC analysis.

**FIGURE 2:**
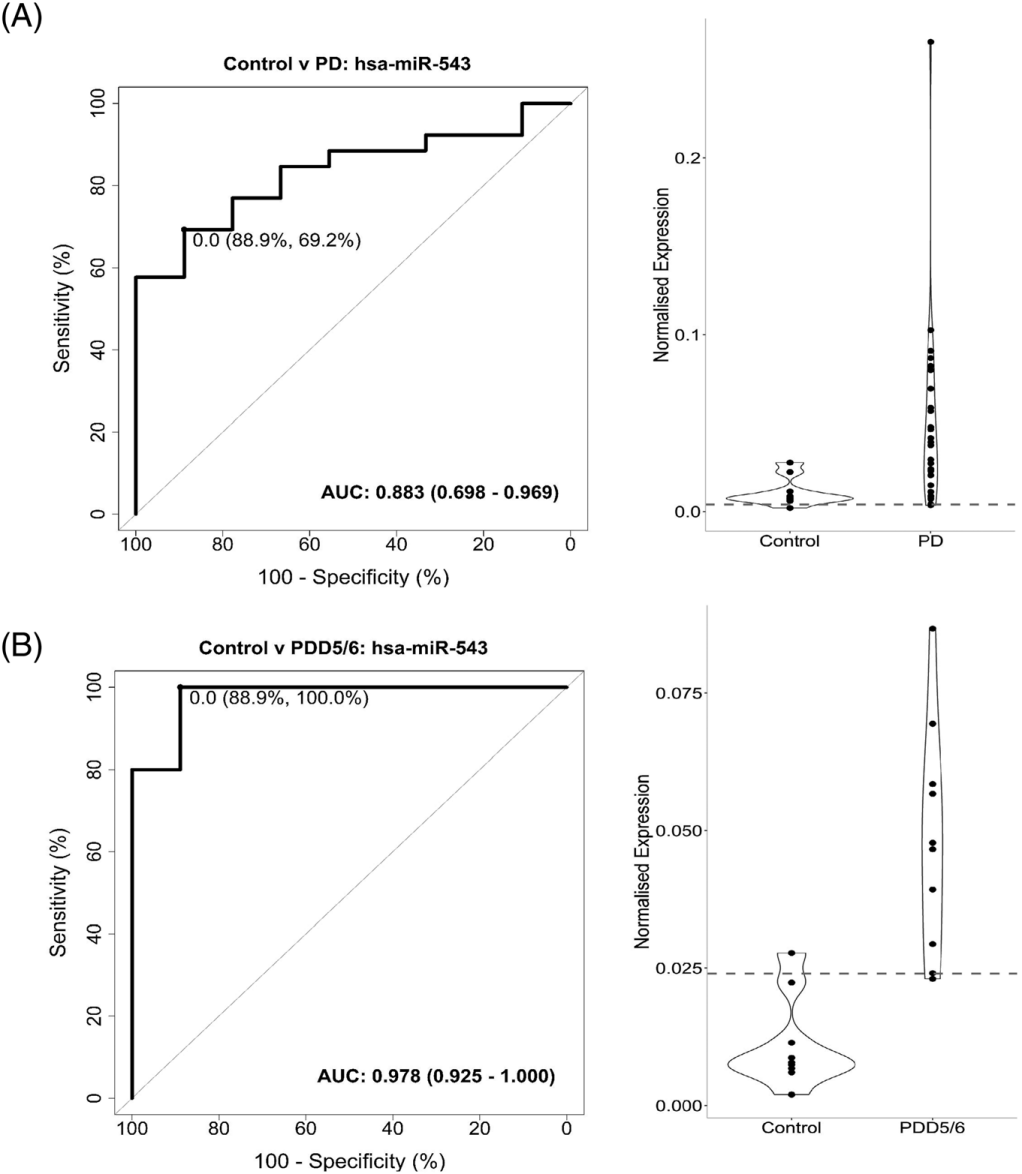
Receiver operating characteristic (ROC) curve analysis and violin plots for PD and PDD5/6. (A) ROC curve analysis showed an 88.9% specificity and 69.2% sensitivity for miR‐543 in discriminating between the control group and all PD groups combined, corresponding to an AUC of 0.883. (B) For the discrimination between the control group and PDD5/6, miR‐543 showed an 88.9% specificity and 100% sensitivity corresponding to an AUC of 0.978. *X* axis ROC curve: 100‐specificity in percentage (%); *Y* axis ROC curve: sensitivity in percentage (%). *X* axis violin plot: groups; *Y* axis violin plot: normalised expression. Control: *n* = 9, PD4: *n* = 6, PD5/6: *n* = 10, PDD5/6: *n* = 10

For miR‐543, the overall diagnostic accuracy of distinguishing between controls and PD demonstrated an AUC of 0.833 with a sensitivity of 69.2% and specificity of 88.9% (Figure 2A). Interestingly, when looking at the accuracy in separate PD groups, miR‐543 showed an AUC of 0.978 with a sensitivity of 100% and specificity of 88.9% for distinguishing between controls and PDD5/6 (Figure 2B). To test the robustness of miR‐543, a permutation analysis (*n* = 30,000) was performed. The permutation analysis demonstrated that miR‐543 performed better than would be expected by chance alone (*p* < 0.05). Finally, miR‐543 did not show high diagnostic accuracy for PD4 and PD5/6 (supporting information Figure S3). For let‐7e‐3p, a low diagnostic accuracy was found for distinguishing between groups, eliminating this miRNA as a possible biomarker for PD (supporting information Figure S4).

### Target prediction of let‐7e‐3p, miR‐424‐3p and miR‐543

To increase our knowledge of the role of let‐7e‐3p, miR‐424‐3p and miR‐543, we continued with further bioinformatic analysis through pathway enrichment analysis. The target genes of the differentially expressed miRNAs were predicted bioinformatically using TargetScan [27]. mRNA targets for let‐7e‐3p were predicted through TargetScan and assessed for enriched pathways (supporting information Table S5). Pathways related to the regulation of synaptic plasticity and modulation of synaptic transmission were among the most significantly enriched pathways for the targets of let‐7e‐3p (supporting information Table S6). For miR‐424‐3p, the predicted mRNA targets (supporting information Table S7) were enriched for pathways including positive regulation of neurogenesis and positive regulation of neuron projection development (supporting information Table S8). For miR‐543, the TargetScan predicted mRNA targets (supporting information Table S9) were enriched for white matter‐related terms such as axonogenesis and axon guidance (Table 2), indicating a possible involvement in the early observed white matter changes in PD. With the observed changes in the expression of miR‐543 in the white matter, the biomarker potential of miR‐543 and the white matter‐related terms in the pathway enrichment, we decided to further focus on miR‐543. Therefore, further determination of the target genes of miR‐543 was done through the validated target database miRTarBase [34]. *SIRT1* was identified as a potential target of miR‐543 and was confirmed as a relevant target in PD through a literature search. Therefore, we hypothesised that miR‐543 might regulate the expression of *SIRT1* and selected miR‐543 and target gene *SIRT1* for subsequent experiments.

**TABLE 2:** Enrichment analysis of target genes of miR‐543. Gene ontology (GO) biological process and molecular function

GO biological process	ID	Gene (*n*)	*p* adjust
Homophilic cell adhesion via plasma membrane adhesion molecules	GO:0007156	54	1.11E‐08
Cell–cell adhesion via plasma‐membrane adhesion molecules	GO:0098742	71	3.57E‐07
**Axonogenesis**	GO:0007409	98	0.000134002
Cell junction assembly	GO:0034329	89	0.000144381
Synapse organisation	GO:0050808	86	0.000793798
**Axon guidance**	GO:0007411	63	0.000924228
Neuron projection guidance	GO:0097485	62	0.000924228
Flavonoid metabolic process	GO:0009812	10	0.001557872
Cell–cell junction organisation	GO:0045216	46	0.014015351
Renal system development	GO:0072001	58	0.021709702
**GO molecular function**	**ID**	**Gene (*n*)**	***p* adjust**
ATPase activity	GO:0016887	84	0.004980871
Small GTPase binding	GO:0031267	80	0.022950198
Ras GTPase binding	GO:0017016	78	0.022950198
Protein‐macromolecule adaptor activity	GO:0030674	51	0.022950198
Phosphoprotein phosphatase activity	GO:0004721	40	0.023065772
Active transmembrane transporter activity	GO:0022804	66	0.024237611
Molecular adaptor activity	GO:0060090	61	0.030692398
Ras guanyl‐nucleotide exchange factor activity	GO:0005088	28	0.033560572
ATPase‐coupled transmembrane transporter activity	GO:0042626	25	0.040582675
Primary active transmembrane transporter activity	GO:0015399	26	0.045309648

### *SIRT1* expression in PD tissue

As *SIRT1* is only a validated target of miR‐543 in organs and tissue outside the brain, we were initially interested in determining the relationship between *SIRT1* and miR‐543 in PD brain tissue. We investigated both the mRNA and protein expression of SIRT1. *SIRT1* mRNA expression was determined through both RNA‐Seq and RT‐qPCR. Although RNA‐Seq showed no dysregulation of *SIRT1* mRNA in either the grey or white matter at any stage of PD, RT‐qPCR was used to validate *SIRT1* mRNA expression in both grey and white matter. The mRNA level of *SIRT1* was shown to be stable across all four groups in both white and grey matter, with only a slight upward trend for PDD5/6 in the white matter (supporting information Figure S5A) and the grey matter (supporting information Figure S5B).

**FIGURE 3:**
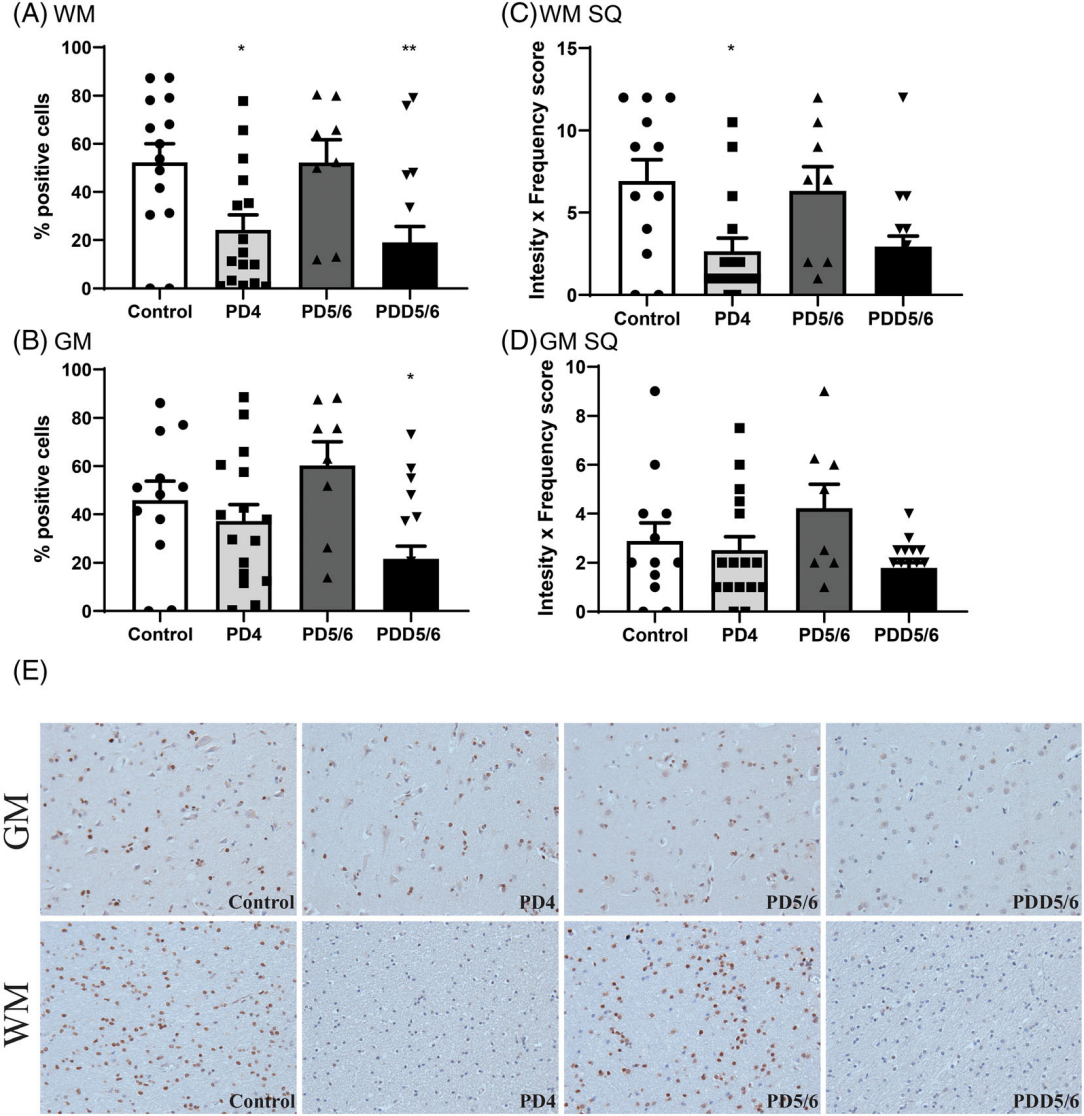
SIRT1 protein expression in grey (GM) and white matter (WM). (A and B) Quantitative analysis of SIRT1 protein expression in the white and grey matter as a measure of percentage positive cells. (C and D) Semi‐quantitative analysis of SIRT1 protein expression in white and grey matter. (E) Immunohistochemical staining of SIRT1 protein controls and 3 PD groups in both grey and white matter. Control: *n* = 10, PD4: *n* = 16, PD5/6: *n* = 8, PDD5/6: *n* = 17. * = *p* < 0.05, ** = *p* < 0.01. Control: *n* = 14, PD4: *n* = 16, PD5/6: *n* = 8, PDD5/6: *n* = 17

More importantly, we looked at the expression of SIRT1 protein by immunohistochemistry, as we predicted that differential expression of miR‐543 would result in a decrease in SIRT1 protein if the gene is a target of miR‐543. To ensure valid results, SIRT1 protein was analysed quantitatively and semi‐quantitatively. After quantification, we found a significantly lower expression of SIRT1 in the white matter in PD4 compared to the control group (Figure 3A/C). Interestingly, we also found downregulation of SIRT1 protein in PDD5/6 in white and grey matter (Figure 3B/D). Immunohistochemical staining for SIRT1 (Figure 3E) showed a clear intensity difference between the control group and PD groups. Moreover, it showed that SIRT1 is expressed in both grey and white matter, whereas in PD without dementia only changes in expression were observed in the white matter.

### *SIRT1* is a direct target of miR‐543 in SH‐SY5Y cells and foetal astrocytes

**FIGURE 4:**
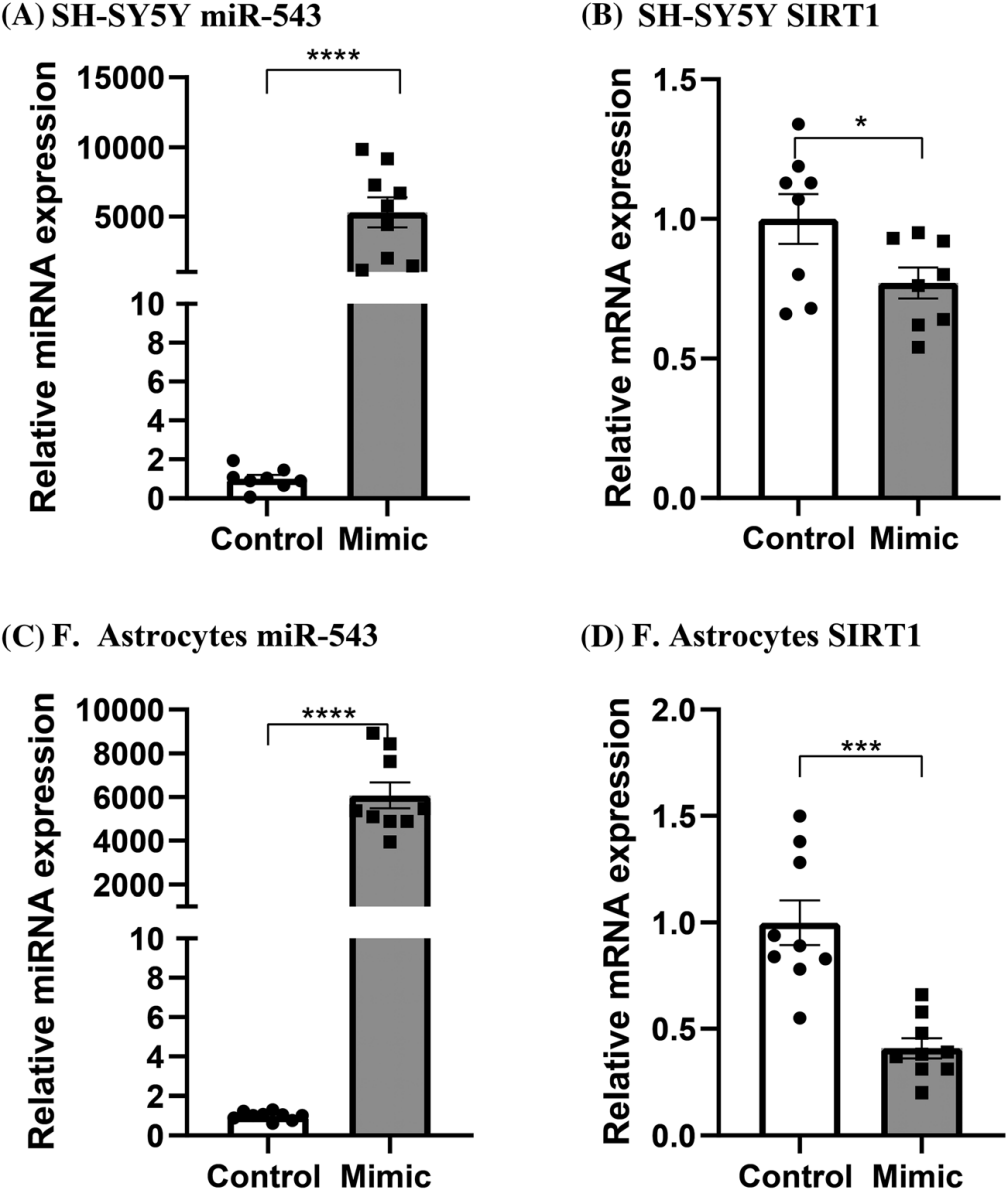
Expression of miR‐543 and *SIRT1* mRNA after transfection with miR‐543 mimic. (A) miR‐543 is increased significantly after transfection with mimic in SH‐SY5Y cells. (B) The expression of *SIRT1* mRNA is significantly decreased after transfection with miR‐543 mimic. (C) In foetal astrocytes, miR‐543 mimic transfection increases the expression of miR‐543 significantly. (D) A strong downregulation of *SIRT1* mRNA is observed after the transfection with miR‐543 mimic. *X* axis: groups, *Y* axis: relative expression compared to control group. Error bars indicate SEM. *p < 0.05, ***p < 0.001, ****p < 0.0001

To further validate that *SIRT1* is a target of miR‐543 in the brain and determine whether this targeting only occurs in the white matter, as tissue experiments indicate, we transfected both SH‐SY5Y cells and foetal astrocytes with a miR‐543 mimic. miR‐543 and *SIRT1* expression was determined by RT‐qPCR. We found that transfection with miR‐543 mimics significantly increases the expression of miR‐543 in both SH‐SY5Y cells and foetal astrocytes (Figure 4A and C, respectively). Furthermore, after transfection with miR‐543, *SIRT1* mRNA was significantly decreased in both SH‐SY5Y cells and foetal astrocytes (Figure 4B and D, respectively). The much stronger downregulation found in foetal astrocytes might suggest that targeting of *SIRT1* by miR‐543 is more pronounced in the white matter compared to the grey matter.

Additionally, to investigate whether the downregulation of mRNA after miR‐543 mimic transfection also leads to the downregulation of SIRT1 protein, we determined the protein expression through western blot. A visible downregulation of protein was observed in foetal astrocytes, but not in SH‐SY5Y cells, indicating that upregulation of miR‐543 downregulates SIRT1 in the white matter but not in the grey matter which is in line with the data from PD tissue (supporting information Figure S6).

## DISCUSSION

Identifying differentially expressed miRNAs in PD increases our knowledge of the underlying mechanisms of the prodromal PD pathology and possibly the early white matter changes. In this study, we found a total of 12 differentially expressed miRNAs. Three of these differentially expressed miRNAs were validated in an independent cohort through RT‐qPCR, with miR‐543 being the only miRNA to show dysregulation in the white matter in early PD. The diagnostic properties of three miRNAs were evaluated, pointing to miR‐543 having a strong biomarker potential in PDD5/6. Functionally characterising miR‐543 identified its potential role in PD‐related white matter changes, with the identification of its putative target *SIRT1*. Upregulation of miR‐543 appears to lead to the downregulation of SIRT1 in patient tissue, as well as astrocyte cell cultures. However, the role of miR‐543 in other PD models and the role of other molecules that may be regulated by miR‐543 have yet to be investigated.

PD is a clinically common age‐dependent disease, and its incidence is increasing as societies around the world continue to shift towards an older demographic. The clinical manifestations of early PD are non‐specific, which makes it hard to identify early intervention points. Therefore, it is necessary to find new effective diagnostic markers and therapeutic targets for PD. A variety of previous studies have shown that miRNAs play important roles in the development and progression of PD [15, 16, 35]. In the present study, it was found that miR‐543 was significantly upregulated in the white matter of patients in the early stage of PD. These findings suggest a potential role for miR‐543 in the observed early white matter changes in PD. More importantly, the regulation of different signalling pathways is likely important in disease progression and miRNAs play a major role in this regulation. As therapeutics using miRNAs are yet to be functional, it is interesting to look further downstream at a singular target of dysregulated miRNAs, in this case, *SIRT1*.

Although there are limited studies identifying miR‐543 as a dysregulated miRNA in PD, a recent study found that the inhibition of miR‐543‐3p can possibly relieve dyskinesia in a PD model through the rescue of glutamate transporter type 1 (*GLT‐1*) expression and function [36]. This is in line with the observed upregulation of miR‐543 that could contribute to PD pathology. Previous studies have found miR‐424 to be dysregulated in PD [37], and this miRNA was reported to be significantly altered in a study in which the expression levels of miR‐424 pre‐ and post‐deep brain stimulation were compared [38]. For let‐7e‐3p, no studies have reported dysregulation in PD. Identification of novel dysregulated miRNAs in PD was possible because of the separation of grey and white matter, as well as different stages of the disease. Although many studies compare control and PD groups overall, the separation of white and grey matter, and early and late stages has allowed for new insights into miRNA dysregulation.

Previously, studies have found miR‐543 to be negatively correlated with *SIRT1* expression in gastric cancer tissues [39]. Moreover, a study focused on insulin resistance showed that overexpression of miR‐543 lowered *SIRT1* mRNA and protein levels in different gastric cell types [40], indicating a negative correlation between miR‐543 and *SIRT1* expression. However, none of these studies focused on the potential role of miR‐543 and its effect on the expression of *SIRT1* in the brain. Therefore, we first looked at the expression of miR‐543 and *SIRT1* in patient tissue in grey and white matter separately to determine whether targeting is cell type specific. Next, by identifying *SIRT1* as a target of miR‐543 in both SH‐SY5Y cells and foetal astrocytes on mRNA level and in foetal astrocytes on protein level, we have provided evidence of a similar relationship between this miRNA and gene in the brain, with this relationship appearing to be most common in the white matter.

*SIRT1*, a member of the sirtuin family, is a nicotinamide adenine dinucleotide (NAD)‐dependent histone deacetylase [41]. Together with other members of the sirtuin family, *SIRT1* has been found to act as a deacetylase for numerous protein targets involved in pathways including axonal degeneration [42]. Therefore, dysregulation of *SIRT1* expression in PD is hypothesised to contribute to PD pathology. Previous studies have shown that the enzymatic activity of SIRT1 is decreased in patients with PD, which may reduce their ability to resist neuronal damage caused by various cytokines, neurotoxins and α‐Syn aggregation [43]. Over the years, *SIRT1* has been described to be possibly protective against the toxic α‐Syn aggregation through the activation of molecular chaperones such as heat shock protein Hsp70. *SIRT1* overexpression studies showed a reduction in α‐Syn aggregates, whereas deletion of *SIRT1* increased α‐Syn aggregation [44]. Furthermore, *SIRT1* was also described to reduce inflammation, apoptosis, and even the activation of astrocytes [45]. Finally, oxidative stress and dysfunction of mitochondria can be caused by the dysregulation of *SIRT1* [43]. Thus, with the upregulation of miR‐543 resulting in the downregulation of *SIRT1*, it can be hypothesised that this influences the neuroprotective role of *SIRT1* in the brain. Considering the fact that both miR‐543 and *SIRT1* seem to be most affected in the white matter, the downstream cascade of SIRT1 downregulation may contribute to the early white matter changes observed in PD. Therefore, while a lot more knowledge is required concerning the role of miR‐543 and *SIRT1* in PD and other neurodegenerative diseases, both can be considered potential neuroprotective therapeutic targets. This would be possible through either the downregulation of miR‐543 in early PD or the upregulation of *SIRT1* on its own. However, for the potential use of miR‐543 as a therapeutic target, it would be necessary to investigate other targets of miR‐543 and how they are regulated in PD. The regulation of *SIRT1* is very promising and is in line with the studies focused on SIRT1 activator resveratrol. A recent study showed that resveratrol displayed neuroprotective efficacy in several animal models of PD [46]. Furthermore, resveratrol can enhance mitochondrial function via SIRT1 pathways [47].

Although we have clearly shown that *SIRT1* is a target of miR‐543, the identification and evaluation of other miRNAs involved in the regulation of this gene deserve further investigation. starBase v2.0 was used to look into this further into this issue and identify miRNAs targeting *SIRT1* [48, 49]. For this search, a very high stringency was used, which meant that more than five CLIP‐seq experiments supported the predicted miRNA target site. This resulted in a total of 29 other miRNAs that have been reported to target *SIRT1* (supporting information Table S10). Moreover, a recent study has shown that miR‐384‐5p targets SIRT1 in mice and SH‐SY5Y cell lines. This study found that this regulation possibly promotes the progression of PD through cell apoptosis. However, as this study was performed in mice and SH‐SY5Y cell lines, the results are mostly applicable to the grey matter [50]. Therefore, the involvement of miR‐543 in the regulation of *SIRT1* in the white matter further supports the role of early white matter changes found in PD.

Although the expression of miR‐543 and *SIRT1* in tissue indicates that miR‐543 only targets *SIRT1* in the early stages of PD in the white matter, miR‐543 was also looked at for its biomarker potential. Initially, it was hypothesised that miR‐543 could be a potential biomarker for PD in general or early PD. Although the overall biomarker potential discriminating the control group from the three PD groups combined, showed promise with an AUC of 0.83, a more interesting finding was discovered when separating the three PD groups. miR‐543 showed strong potential as a biomarker for PDD5/6 (AUC: 0.978). This result suggests that while miR‐543 is dysregulated in PD without dementia, it can also be implicated in PD with dementia. As this biomarker was determined in CSF at the final stage of the disease, it would be interesting to look further into the biomarker potential of miR‐543. The expression patterns and prognostic accuracy of miR‐543 should be investigated in more readily available biofluids such as blood/serum, and this could lead to the development of a simple, less‐invasive biomarker for cognitive impairment in PD.

## CONCLUSION

In conclusion, analysis of differentially expressed miRNAs in PD has led to the identification of miR‐543 as a dysregulated miRNA. Furthermore, miR‐543 is identified as a regulator of *SIRT1* in the white matter of the brain, which possibly implicates both the miRNA and the gene in the early white matter changes in PD, and miR‐543 has also shown potential diagnostic accuracy for dementia in PD patients. With the discovery of a potential biomarker, it is important that future studies investigate the possibility of applying biomarker research in the clinic. The work presented here shows a possible framework that could lead to the discovery of dysregulated pathways in PD and the identification of novel therapeutic targets. Nonetheless, further investigation is still required to establish the role of miR‐543 and *SIRT1* in PD and miR‐543 as potential biomarker or possible therapeutic target.

## CONFLICTS OF INTEREST

The authors report no conflict of interest.

## ETHICS STATEMENT

The research presented in this manuscript was in accordance with the Declaration of Helsinki and the Amsterdam UMC Research Code provided by the Medical Ethics Committee, and the study was approved by the local ethical committees of all participating medical centres.

## AUTHOR CONTRIBUTIONS

E. A and J. D. M conceptualised the project. Material preparation was performed by J. A and A. I. J. D. M and M. S analysed the RNA sequencing data and further bioinformatic analyses. M. S and L. M designed and performed IHC experiments, qPCR and RT‐qPCR experiments, and all in vitro experiments. M. S drafted the first manuscript and prepared the figures, and all authors commented on previous versions of the manuscript. All authors read and approved the final manuscript.

### PEER REVIEW

The peer review history for this article is available at https://publons.com/publon/10.1111/nan.12864.

## Supporting information


**Supplementary Figure 1.** Volcano plot displaying the differentially expressed miRNAs in PD compared to controls (adjusted p‐value <0.05). The x‐axis shows the miRNA expression levels in Log2FoldChange (Log_2_FC) and the y‐axis shows the significance levels of the miRNA expression in ‐log_10_ (Adj. p‐value). In total 9 miRNAs were found to be under expressed (blue) and 3 miRNAs were found to be overexpressed (red).
**Figure S2.** Expression of cell type markers in RNA sequencing cohort using RT‐qPCR. A. Microglia marker (IBA1) expression in white matter (WM). B. Astrocytes marker (GFAP) in white matter. C. IBA1 expression in grey matter (GM). D. GFAP expression in grey matter. E. Neuron marker (NeuN) expression grey matter for the samples used in the RNA sequencing cohort. Expression is shown as N0 ratio, comparing control and PD group. No differential expression was found between any control and PD groups after Mann–Whitney non‐parametric t‐tests.
**Figure S3.** Receiver operating characteristic (ROC) curve analysis and violin plots of miR‐543 for PD4 and PD5/6. A. ROC curve analysis showed a 66.7% specificity and 83.3% sensitivity for miR‐543 in discriminating between the control group and the PD4 group, corresponding to an AUC of 0.759. B. For the discrimination between the control group and PD5/6, miR‐543 showed a 100% specificity and 40% sensitivity corresponding to an AUC of 0.733. X‐axis ROC curve: 100‐specificity in percentage (%); Y‐axis ROC curve: sensitivity in percentage (%). X‐axis violin plot: groups; Y‐axis violin plot: Normalized expression. Control: n = 9, PD4: n = 6, PD5/6: n = 10, PDD5/6: n = 10.
**Figure S4.** Receiver operating characteristic (ROC) curve analysis and violin plots of let‐7e‐3p for all PD groups combined, PD4, PD5/6 and PDD5/6. A. ROC curve analysis showed a 77.8% specificity and 46.2% sensitivity for let‐7e‐3p in discriminating between the control group and all PD groups combined, corresponding to an AUC of 0.487. B. For the discrimination between the control group and PD4, let‐7e‐3p showed a 77.8% specificity and 66.7% sensitivity corresponding to an AUC of 0.630. C. A ROC curve analysis showed 77.8% specificity and 50% sensitivity for let‐7e‐3p in discriminating between the control group and PD5/6, corresponding to an AUC of 0.489. D. For the discrimination between the control group and PDD5/6, let‐7e‐3p showed a 77.8% specificity and 50% sensitivity corresponding to an AUC of 0.556. X‐axis ROC curve: 100‐specificity in percentage (%); Y‐axis ROC curve: sensitivity in percentage (%). X‐axis violin plot: groups; Y‐axis violin plot: Normalized expression. Control: n = 9, PD4: n = 6, PD5/6: n = 10, PDD5/6: n = 10.
**Figure S5.** *SIRT1* mRNA expression in white and grey matter in controls and across PD groups. A. White matter expression of *SIRT1* mRNA in the control group and 3 PD groups. B. Grey matter expression of *SIRT1* mRNA in the control group and 3 PD groups. X‐axis: relative mRNA expression relative to controls. Y‐axis: groups. Error bars indicate SEM. Control: n = 10, PD4: n = 11, PD5/6: n = 9, PDD5/6: n = 19.
**Figure S6.** Western blot (WB) of SIRT1 in foetal astrocytes and SH‐SY5Y cells. Glyceraldehyde‐3‐phosphate dehydrogenase (GAPDH) was used as loading control. Blots for GAPDH were developed simultaneously for foetal astrocytes and SH‐SY5Y cells, SIRT1 in foetal astrocytes was developed using ECL plus and SIRT1 in SH‐SY5Y cells was developed using ECL. All blots were cropped and merged into one figure.
**Supplementary Table 1.** Clinical summary of controls, PD4, PD5/6 and PDD5/6, with techniques the samples were used for.
**Supplementary Table 2.** Primers and sequences used in qPCR.
**Supplementary Table 3.** List of primers used for TaqMan RT‐qPCR.
**Supplementary Table 4**. List of antibodies used in experiments.
**Supplementary Table 5.** Top 30 predicted mRNA targets of let‐7e‐3p from TargetScan
**Supplementary Table 6.** Enrichment analysis of target genes of let‐7e‐3p. Gene ontology biological process and molecular function.
**Supplementary Table 7.** Top 30 predicted mRNA targets of miR‐424‐3p from TargetScan
**Supplementary Table 8.** Enrichment analysis of target genes of miR‐424‐3p. Gene ontology biological process and molecular function.
**Supplementary Table 9.** Top 30 predicted mRNA targets of miR‐543 from TargetScan
**Supplementary Table 10.** miRNAs targeting SIRT1

## Data Availability

The data that support the findings of this study are available from the corresponding author upon reasonable request.
