# Supplementary material for: Dysregulation of miR‐543 in Parkinson's disease: Impact on the neuroprotective gene SIRT1
Source: Neuropathol Appl Neurobiol. 2022 Nov 26;49(1):e12864. doi: 10.1111/nan.12864 (PMC10100056; doi:10.1111/nan.12864)
Supplement: Supplementary file 1 — Supplementary Figure 1. Volcano plot displaying the differentially expressed miRNAs in PD compared to controls (adjusted p‐value <0.05). The x‐axis shows the miRNA expression levels in Log2FoldChange (Log2FC) and the y‐axis shows the significance levels of the miRNA expression in ‐log10 (Adj. p‐value). In total 9 miRNAs were found to be under expressed (blue) and 3 miRNAs were found to be overexpressed (red). Figure S2. Expression of cell type markers in RNA sequencing cohort using RT‐qPCR. A. Microglia marker (IBA1) expression in white matter (WM). B. Astrocytes marker (GFAP) in white matter. C. IBA1 expression in grey matter (GM). D. GFAP expression in grey matter. E. Neuron marker (NeuN) expression grey matter for the samples used in the RNA sequencing cohort. Expression is shown as N0 ratio, comparing control and PD group. No differential expression was found between any control and PD groups after Mann–Whitney non‐parametric t‐tests. Figure S3. Receiver operating characteristic (ROC) curve analysis and violin plots of miR‐543 for PD4 and PD5/6. A. ROC curve analysis showed a 66.7% specificity and 83.3% sensitivity for miR‐543 in discriminating between the control group and the PD4 group, corresponding to an AUC of 0.759. B. For the discrimination between the control group and PD5/6, miR‐543 showed a 100% specificity and 40% sensitivity corresponding to an AUC of 0.733. X‐axis ROC curve: 100‐specificity in percentage (%); Y‐axis ROC curve: sensitivity in percentage (%). X‐axis violin plot: groups; Y‐axis violin plot: Normalized expression. Control: n = 9, PD4: n = 6, PD5/6: n = 10, PDD5/6: n = 10. Figure S4. Receiver operating characteristic (ROC) curve analysis and violin plots of let‐7e‐3p for all PD groups combined, PD4, PD5/6 and PDD5/6. A. ROC curve analysis showed a 77.8% specificity and 46.2% sensitivity for let‐7e‐3p in discriminating between the control group and all PD groups combined, corresponding to an AUC of 0.487. B. For the discrimination between [file NAN-49-0-s001.docx]

**Supplementary data**


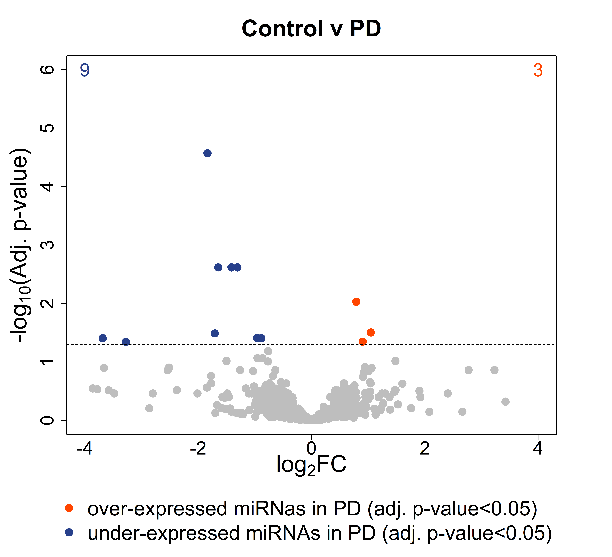


**Supplementary figure 1.** Volcano plot displaying the differentially expressed miRNAs in PD compared to controls (adjusted p-value <0.05). The x-axis shows the miRNA expression levels in Log2FoldChange (Log_2_FC) and the y-axis shows the significance levels of the miRNA expression in -log_10_ (Adj. p-value). In total 9 miRNAs were found to be under expressed (blue) and 3 miRNAs were found to be overexpressed (red).

**Supplementary figure 2**. Expression of cell type markers in RNA sequencing cohort using RT-qPCR. A. Microglia marker (IBA1) expression in white matter (WM). B. Astrocytes marker (GFAP) in white matter. C. IBA1 expression in grey matter (GM). D. GFAP expression in grey matter. E. Neuron marker (NeuN) expression grey matter for the samples used in the RNA sequencing cohort. Expression is shown as N0 ratio, comparing control and PD group. No differential expression was found between any control and PD groups after Mann-Whitney non-parametric t-tests.


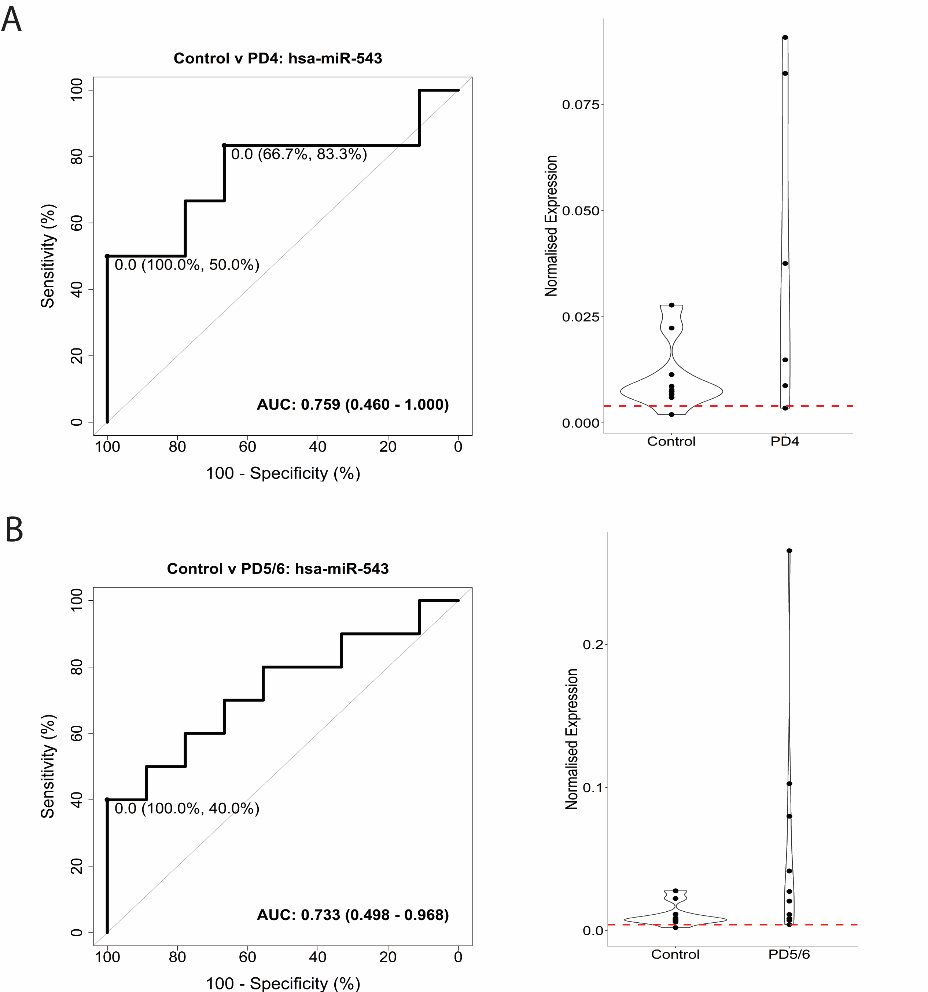


**Supplementary figure 3.** Receiver operating characteristic (ROC) curve analysis and violin plots of miR-543 for PD4 and PD5/6 . A. ROC curve analysis showed a 66.7% specificity and 83.3% sensitivity for miR-543 in discriminating between the control group and the PD4 group, corresponding to an AUC of 0.759. B. For the discrimination between the control group and PD5/6, miR-543 showed a 100% specificity and 40% sensitivity corresponding to an AUC of 0.733. X-axis ROC curve: 100-specificity in percentage (%); Y-axis ROC curve: sensitivity in percentage (%). X-axis violin plot: groups; Y-axis violin plot: Normalized expression. Control: n=9, PD4: n =6, PD5/6: n=10, PDD5/6: n=10.


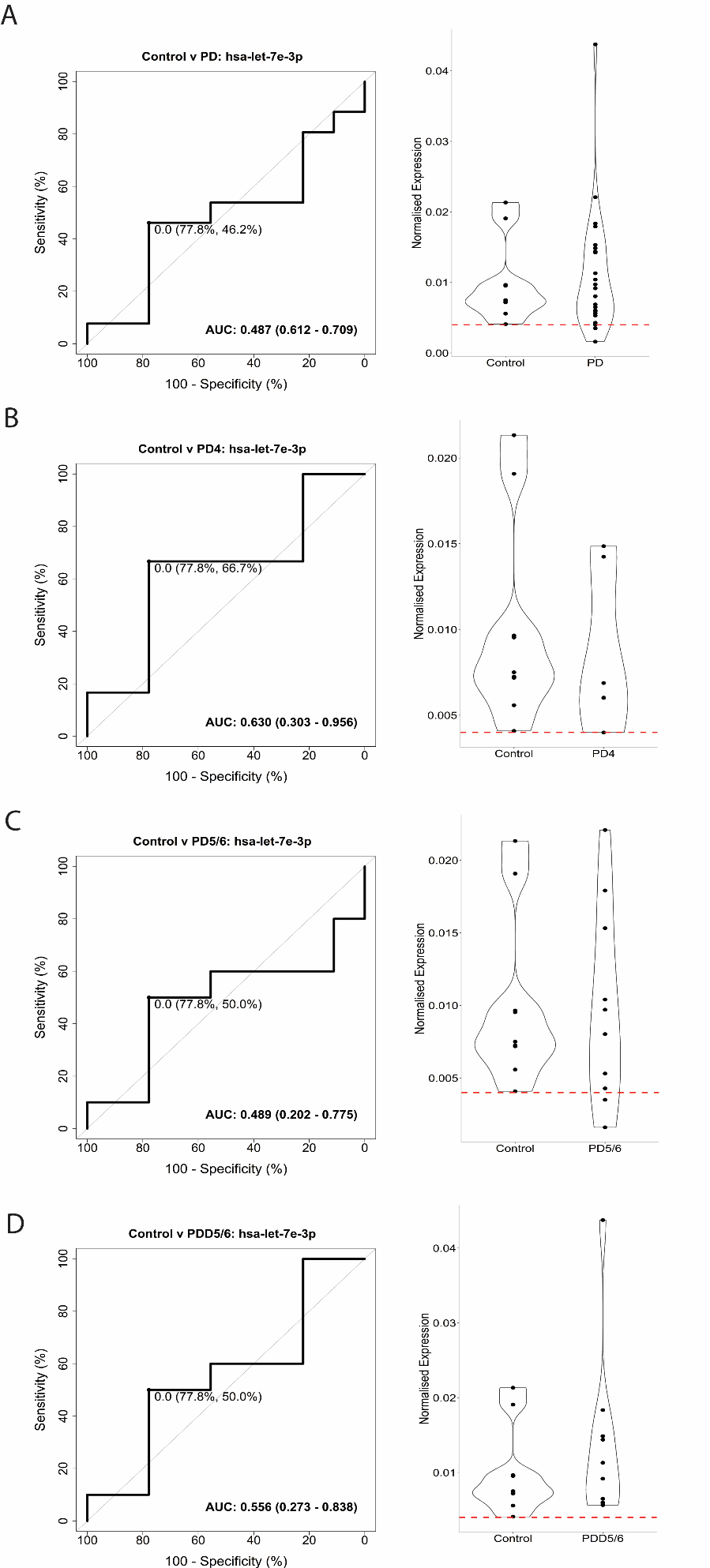


**Supplementary figure 4.** Receiver operating characteristic (ROC) curve analysis and violin plots of let-7e-3p for all PD groups combined, PD4, PD5/6 and PDD5/6. A. ROC curve analysis showed a 77.8% specificity and 46.2% sensitivity for let-7e-3p in discriminating between the control group and all PD groups combined, corresponding to an AUC of 0.487. B. For the discrimination between the control group and PD4, let-7e-3p showed a 77.8% specificity and 66.7% sensitivity corresponding to an AUC of 0.630. C. A ROC curve analysis showed 77.8% specificity and 50% sensitivity for let-7e-3p in discriminating between the control group and PD5/6, corresponding to an AUC of 0.489. D. For the discrimination between the control group and PDD5/6, let-7e-3p showed a 77.8% specificity and 50% sensitivity corresponding to an AUC of 0.556. X-axis ROC curve: 100-specificity in percentage (%); Y-axis ROC curve: sensitivity in percentage (%). X-axis violin plot: groups; Y-axis violin plot: Normalized expression. Control: n=9, PD4: n =6, PD5/6: n=10, PDD5/6: n=10.

**Supplementary figure 5.** *SIRT1* mRNA expression in white and grey matter in controls and across PD groups. A. White matter expression of *SIRT1* mRNA in the control group and 3 PD groups. B. Grey matter expression of *SIRT1* mRNA in the control group and 3 PD groups. X-axis: relative mRNA expression relative to controls. Y-axis: groups. Error bars indicate SEM. Control: n=10, PD4: n =11, PD5/6: n=9, PDD5/6: n=19.


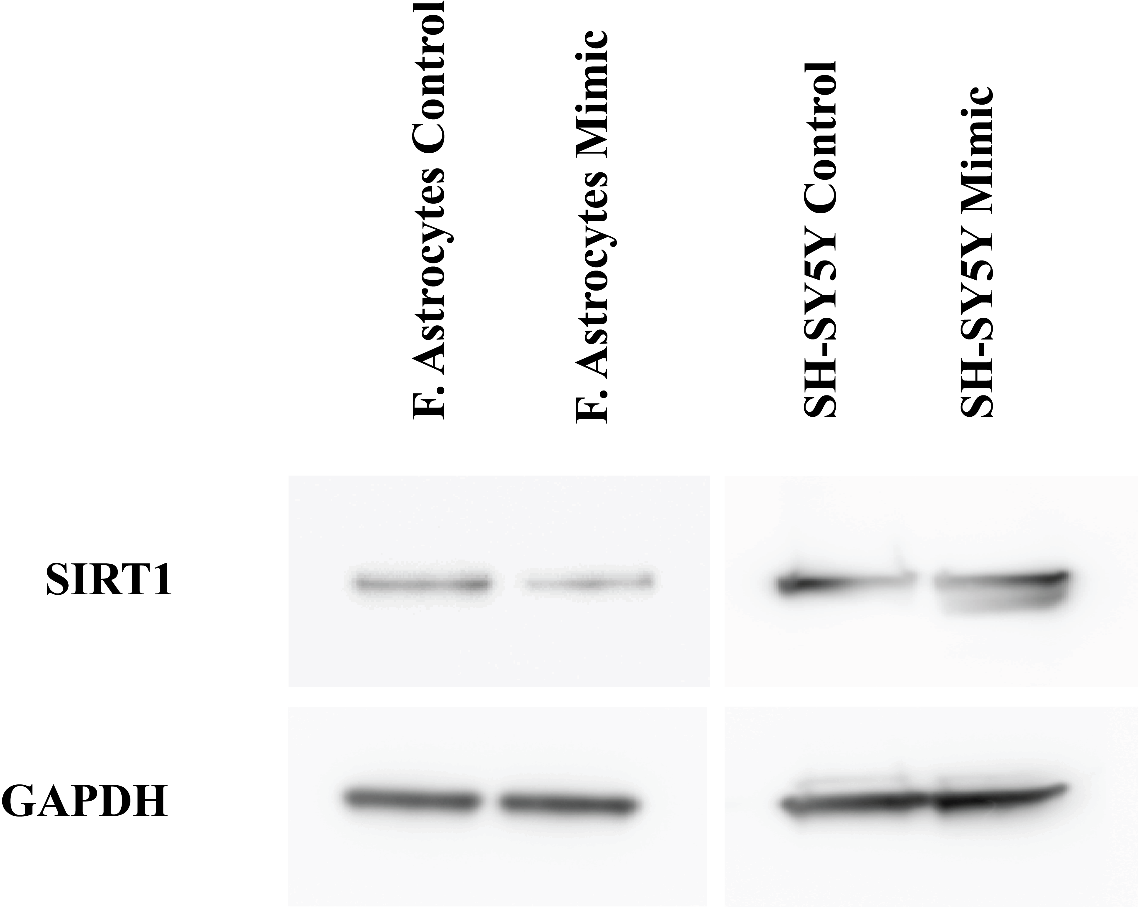


**Supplementary figure 6.** Western blot (WB) of SIRT1 in foetal astrocytes and SH-SY5Y cells. Glyceraldehyde-3-phosphate dehydrogenase (GAPDH) was used as loading control. Blots for GAPDH were developed simultaneously for foetal astrocytes and SH-SY5Y cells, SIRT1 in foetal astrocytes was developed using ECL plus and SIRT1 in SH-SY5Y cells was developed using ECL. All blots were cropped and merged into one figure.

**Supplementary table 1.** Clinical summary of controls, PD4, PD5/6 and PDD5/6, with techniques the samples were used for.

| **ID** | **Group** | **Sex** | **Age (y)** | **Used for** |
| --- | --- | --- | --- | --- |
| 1 | Control | m | 102 | CSF, miRNA validation, qPCR, immunohistochemistry |
| 2 | Control | m | 91 | CSF, miRNA validation, qPCR, immunohistochemistry |
| 3 | Control | f | 71 | CSF, miRNA validation, qPCR |
| 4 | Control | m | 86 | CSF, miRNA validation, qPCR |
| 5 | Control | m | 88 | CSF, miRNA validation, qPCR, immunohistochemistry |
| 6 | Control | f | 102 | CSF, miRNA validation, qPCR, immunohistochemistry |
| 7 | Control | f | 82 | CSF, miRNA validation, qPCR, immunohistochemistry |
| 8 | Control | f | 95 | CSF, miRNA validation, qPCR, immunohistochemistry |
| 9 | Control | m | 87 | CSF, miRNA validation, qPCR |
| 10 | Control | f | 87 | GM/WM RNA sequencing, qPCR Immunohistochemistry |
| 11 | Control | m | 88 | GM/WM RNA sequencing, Immunohistochemistry |
| 12 | Control | f | 81 | GM/WM RNA sequencing, Immunohistochemistry |
| 13 | Control | f | 92 | GM/WM RNA sequencing, Immunohistochemistry |
| 14 | Control | f | 78 | WM RNA sequencing, Immunohistochemistry |
| 15 | Control | m | 92 | GM/WM RNA sequencing, Immunohistochemistry |
| 16 | Control | f | 82 | Immunohistochemistry |
| 17 | Control | f | 75 | GM/WM RNA sequencing, Immunohistochemistry |
| 18 | Control | m | 95 | GM/WM RNA sequencing |
| 19 | Control | f | 91 | GM/WM RNA sequencing |
| 20 | PD4 | f | 59 | CSF, miRNA validation, qPCR, immunohistochemistry |
| 21 | PD4 | m | 57 | CSF, miRNA validation, qPCR, immunohistochemistry |
| 22 | PD4 | f | 81 | CSF, miRNA validation, qPCR, immunohistochemistry |
| 23 | PD4 | m | 75 | CSF, miRNA validation, qPCR, immunohistochemistry |
| 24 | PD4 | f | 68 | CSF, miRNA validation, qPCR, immunohistochemistry |
| 25 | PD4 | m | 86 | CSF, miRNA validation, qPCR, immunohistochemistry |
| 26 | PD4 | f | 77 | GM RNA sequencing, qPCR Immunohistochemistry |
| 27 | PD4 | m | 74 | GM/WM RNA sequencing, qPCR, Immunohistochemistry |
| 28 | PD4 | m | 66 | GM RNA sequencing, Immunohistochemistry |
| 29 | PD4 | m | 86 | GM/WM RNA sequencing, qPCR, Immunohistochemistry |
| 30 | PD4 | m | 83 | GM RNA sequencing, Immunohistochemistry |
| 31 | PD4 | f | 80 | GM/WM RNA sequencing, qPCR, Immunohistochemistry |
| 32 | PD4 | f | 82 | GM RNA sequencing, Immunohistochemistry |
| 33 | PD4 | f | 71 | GM/WM RNA sequencing, qPCR, Immunohistochemistry |
| 34 | PD4 | m | 86 | GM/WM RNA sequencing, Immunohistochemistry |
| 35 | PD4 | f | 86 | GM/WM RNA sequencing, Immunohistochemistry |
| 36 | PD5/6 | m | 81 | CSF, miRNA validation, qPCR, immunohistochemistry |
| 37 | PD5/6 | f | 86 | CSF, miRNA validation, qPCR, immunohistochemistry |
| 38 | PD5/6 | m | 56 | CSF, miRNA validation, qPCR, immunohistochemistry |
| 39 | PD5/6 | m | 69 | CSF, miRNA validation, qPCR, immunohistochemistry |
| 40 | PD5/6 | m | 82 | CSF, miRNA validation, qPCR |
| 41 | PD5/6 | f | 66 | CSF, miRNA validation, qPCR, immunohistochemistry |
| 42 | PD5/6 | m | 80 | CSF, miRNA validation, qPCR, immunohistochemistry |
| 43 | PD5/6 | f | 67 | CSF, miRNA validation, qPCR, immunohistochemistry |
| 44 | PD5/6 | m | 74 | CSF, miRNA validation, qPCR, immunohistochemistry |
| 45 | PDD5/6 | m | 72 | CSF, miRNA validation, qPCR, immunohistochemistry |
| 46 | PDD5/6 | f | 86 | CSF, miRNA validation, qPCR |
| 47 | PDD5/6 | f | 80 | CSF, miRNA validation, qPCR, immunohistochemistry |
| 48 | PDD5/6 | f | 81 | CSF, miRNA validation, qPCR, immunohistochemistry |
| 49 | PDD5/6 | m | 67 | CSF, miRNA validation, qPCR, immunohistochemistry |
| 50 | PDD5/6 | m | 77 | CSF, miRNA validation, qPCR, immunohistochemistry |
| 51 | PDD5/6 | m | 86 | CSF, miRNA validation, qPCR, immunohistochemistry |
| 52 | PDD5/6 | f | 76 | CSF, miRNA validation, qPCR, immunohistochemistry |
| 53 | PDD5/6 | m | 80 | CSF, miRNA validation, qPCR, immunohistochemistry |
| 54 | PDD5/6 | f | 83 | CSF, miRNA validation, qPCR, immunohistochemistry |
| 55 | PDD5/6 | m | 71 | GM/WM RNA sequencing, qPCR, Immunohistochemistry |
| 56 | PDD5/6 | m | 88 | GM/WM RNA sequencing, qPCR, Immunohistochemistry |
| 57 | PDD5/6 | m | 83 | GM/WM RNA sequencing, qPCR, Immunohistochemistry |
| 58 | PDD5/6 | m | 80 | GM/WM RNA sequencing, qPCR, Immunohistochemistry |
| 59 | PDD5/6 | f | 70 | GM/WM RNA sequencing, qPCR, Immunohistochemistry |
| 60 | PDD5/6 | m | 80 | GM/WM RNA sequencing, qPCR, Immunohistochemistry |
| 61 | PDD5/6 | m | 74 | GM/WM RNA sequencing, qPCR, Immunohistochemistry |
| 62 | PDD5/6 | m | 70 | GM RNA sequencing, Immunohistochemistry |
| 63 | PDD5/6 | f | 81 | WM RNA sequencing, Immunohistochemistry |
| 64 | PDD5/6 | m | 81 | GM/WM RNA sequencing, qPCR, Immunohistochemistry |

**Abbreviations:** CSF: cerebral spinal fluid, f: female, m: male, GM: gray matter, WM: white matter

**Supplementary table 2.** Primers and sequences used in qPCR.

| **Gene name** | **Full name** | **Sequence designed** |
| --- | --- | --- |
| SIRT1 | Sirtuin 1 | **F**: TGTACGACGAAGACGACGAC  **R**: TTCATCACCGAACAGAAGGTT |
| EF1a | Elongation factor 1 alpha | **F:** ATCCACCTTTGGGTCGCTTT  **R:** CCGCAACTGTCTGTCTCATATCAC |
| IBA1 | Ionized calcium-binding adapter molecule 1 | **F:** CCAAACCAGGGATTTACAGG  **R:** CGTCTAGGAATTGCTTGTTGATCT |
| GFAP | Glial fibrillary acidic protein | **F:** GCCACCTACAGGAAGCTGCT  **R:** GGGAATGGTGATCCGGTTCT |
| NEUN | Neuronal nuclear protein | **F:** GCAGACCTCACCTCCCACTA  **R:** AAAATGGTGGCAGAATTTCCT |

**Supplementary table 3**. List of primers used for TaqMan RT-qPCR

| **miRNA name** | **Company** | **Assay ID** |
| --- | --- | --- |
| hsa-let-7e-3p | ThermoFisher | 002407 |
| hsa-miR-424-3p | ThermoFisher | 002309 |
| hsa-miR-543 | ThermoFisher | 002376 |
| RNU6B | ThermoFisher | 001093 |
| cel-miR-39 | ThermoFisher | 000200 |

**Supplementary table** **4**. List of antibodies used in experiments.

| **Gene name** | **Host** | **Clonality** | **Dilution factor** | **Company** | **Catalog nr.** |
| --- | --- | --- | --- | --- | --- |
| Anti-SIRT1 | Mouse | Monoclonal | IHC: 1:200  WB: 1:1000 | Abcam | Ab110304 |
| Anti-GAPDH | Rabbit | Polyclonal | WB: 1:2000 | Sigma-Aldrich | G9545 |
| Anti-mouse immunoglobin-HRP | Goat | Polyclonal | WB: 1:10000 | DAKO | P044701 |
| Anti-rabbit immunoglobin-HRP | Goat | Polyclonal | WB: 1:10000 | DAKO | P044801 |

**Supplementary table 5.** Top 30 predicted mRNA targets of let-7e-3p from TargetScan

| **Target gene** | **Gene name** |
| --- | --- |
| CDNF | cerebral dopamine neurotrophic factor |
| SRP19 | signal recognition particle 19kDa |
| ISCA2 | iron-sulfur cluster assembly 2 |
| ZNF90 | zinc finger protein 90 |
| AC022498.1 | Uncharacterized protein |
| A4GNT | alpha-1,4-N-acetylglucosaminyltransferase |
| FUBP3 | far upstream element (FUSE) binding protein 3 |
| TMEM163 | transmembrane protein 163 |
| ZCCHC6 | zinc finger, CCHC domain containing 6 |
| CT62 | cancer/testis antigen 62 |
| GREM2 | gremlin 2, DAN family BMP antagonist |
| RAB2B | RAB2B, member RAS oncogene family |
| PHOX2B | paired-like homeobox 2b |
| NUP37 | nucleoporin 37kDa |
| SH3BP5 | SH3-domain binding protein 5 (BTK-associated) |
| FAM230A | family with sequence similarity 230, member A |
| AL139099.1 | Full-length cDNA clone CS0DK012YO09 of HeLa cells of Homo sapiens (human); Uncharacterized protein |
| SPOPL | speckle-type POZ protein-like |
| C21orf54 | chromosome 21 open reading frame 54 |
| NDUFC1 | NADH dehydrogenase (ubiquinone) 1, subcomplex unknown, 1, 6kDa |
| CSNK2A2 | casein kinase 2, alpha prime polypeptide |
| LSR | lipolysis stimulated lipoprotein receptor |
| PHC1 | polyhomeotic homolog 1 (Drosophila) |
| ZNF503 | zinc finger protein 503 |
| RP1-241P17.4 | Uncharacterized protein |
| NUP62CL | nucleoporin 62kDa C-terminal like |
| EXOC8 | exocyst complex component 8 |
| EIF3E | eukaryotic translation initiation factor 3, subunit E |
| IMPG1 | interphotoreceptor matrix proteoglycan 1 |
| SUMO2 | small ubiquitin-like modifier 2 |

**Supplementary table 6.** Enrichment analysis of target genes of let-7e-3p. Gene ontology biological process and molecular function.

| **GO biological process** | **ID** | **Gene (n)** | **p adjust** |
| --- | --- | --- | --- |
| regulation of cell morphogenesis | GO:0022604 | 158 | 1.13E-05 |
| covalent chromatin modification | GO:0016569 | 152 | 1.13E-05 |
| axonogenesis | GO:0007409 | 152 | 1.13E-05 |
| histone modification | GO:0016570 | 148 | 1.13E-05 |
| synapse organization | GO:0050808 | 137 | 3.16E-05 |
| dendrite morphogenesis | GO:0048813 | 57 | 0.000223276 |
| regulation of cell morphogenesis involved in differentiation | GO:0010769 | 100 | 0.000630167 |
| regulation of synaptic plasticity | GO:0048167 | 68 | 0.000706756 |
| dendrite development | GO:0016358 | 82 | 0.001357223 |
| modulation of chemical synaptic transmission | GO:0050804 | 134 | 0.001693428 |
| **GO molecular function** | **ID** | **Gene (n)** | **p adjust** |
| actin binding | GO:0003779 | 143 | 5.53E-05 |
| guanyl-nucleotide exchange factor activity | GO:0005085 | 73 | 0.01292717 |

**Supplementary table 7.** Top 30 predicted mRNA targets of miR-424-3p from TargetScan

| **Target gene** | **Gene name** |
| --- | --- |
| HNRNPA0 | heterogeneous nuclear ribonucleoprotein A0 |
| RHOXF2 | Rhox homeobox family, member 2 |
| TXNL4A | thioredoxin-like 4A |
| AL078585.1 | Uncharacterized protein; cDNA FLJ58069 |
| TMEM70 | transmembrane protein 70 |
| TEX30 | testis expressed 30 |
| CREB3L4 | cAMP responsive element binding protein 3-like 4 |
| EID3 | EP300 interacting inhibitor of differentiation 3 |
| RNF13 | ring finger protein 13 |
| AC027763.2 | Uncharacterized protein |
| SLC26A5 | solute carrier family 26 (anion exchanger), member 5 |
| OAF | OAF homolog (Drosophila) |
| TCEAL1 | transcription elongation factor A (SII)-like 1 |
| RGS21 | regulator of G-protein signaling 21 |
| RHOXF2B | Rhox homeobox family, member 2B |
| THBS4 | thrombospondin 4 |
| FAM122B | family with sequence similarity 122B |
| WIF1 | WNT inhibitory factor 1 |
| BTG1 | B-cell translocation gene 1, anti-proliferative |
| EIF5A | eukaryotic translation initiation factor 5A |
| C11orf52 | chromosome 11 open reading frame 52 |
| ZNF17 | zinc finger protein 17 |
| KLHDC1 | kelch domain containing 1 |
| KCTD18 | potassium channel tetramerization domain containing 18 |
| CSAG1 | chondrosarcoma associated gene 1 |
| EXOSC8 | exosome component 8 |
| GLOD4 | glyoxalase domain containing 4 |
| EIF5AL1 | eukaryotic translation initiation factor 5A-like 1 |
| LACTB2 | lactamase, beta 2 |
| SMIM15 | small integral membrane protein 15 |

**Supplementary table 8.** Enrichment analysis of target genes of miR-424-3p. Gene ontology biological process and molecular function.

| **GO biological process** | **ID** | **Gene (n)** | **p adjust** |
| --- | --- | --- | --- |
| columnar/cuboidal epithelial cell development | GO:0002066 | 25 | 0.002839043 |
| columnar/cuboidal epithelial cell differentiation | GO:0002065 | 36 | 0.010843717 |
| forebrain development | GO:0030900 | 86 | 0.010843717 |
| positive regulation of neuron differentiation | GO:0045666 | 83 | 0.012734627 |
| positive regulation of neurogenesis | GO:0050769 | 101 | 0.012734627 |
| pyruvate metabolic process | GO:0006090 | 41 | 0.012880589 |
| protein deubiquitination | GO:0016579 | 65 | 0.013741784 |
| protein modification by small protein removal | GO:0070646 | 68 | 0.013741784 |
| inner ear receptor cell differentiation | GO:0060113 | 21 | 0.014071359 |
| positive regulation of cell projection organization | GO:0031346 | 84 | 0.014212006 |
| **GO molecular function** | **ID** | **Gene (n)** | **p adjust** |
| thiol-dependent ubiquitin-specific protease activity | GO:0004843 | 39 | 6.81E-05 |
| ubiquitinyl hydrolase activity | GO:0101005 | 39 | 6.81E-05 |
| omega peptidase activity | GO:0008242 | 40 | 0.000362124 |
| RNA polymerase II-specific DNA-binding transcription factor binding | GO:0061629 | 62 | 0.019429154 |
| cytoskeletal anchor activity | GO:0008093 | 11 | 0.019429154 |
| nuclear hormone receptor binding | GO:0035257 | 38 | 0.023050693 |
| phospholipid binding | GO:0005543 | 93 | 0.038111448 |
| phosphatidylinositol binding | GO:0035091 | 59 | 0.038111448 |
| DNA-binding transcription factor binding | GO:0140297 | 74 | 0.038111448 |
| nuclear receptor binding | GO:0016922 | 28 | 0.043569489 |

**Supplementary table 9.** Top 30 predicted mRNA targets of miR-543 from TargetScan

| **Target gene** | **Gene name** |
| --- | --- |
| CYP3A5 | cytochrome P450, family 3, subfamily A, polypeptide 5 |
| NANP | N-acetylneuraminic acid phosphatase |
| FBXO34 | F-box protein 34 |
| TMEM203 | transmembrane protein 203 |
| TAF13 | TAF13 RNA polymerase II, TATA box binding protein (TBP)-associated factor, 18kDa |
| TNFSF11 | tumor necrosis factor (ligand) superfamily, member 11 |
| FBXO47 | F-box protein 47 |
| CYB5R4 | cytochrome b5 reductase 4 |
| FMNL2 | formin-like 2 |
| KIN | KIN, antigenic determinant of recA protein homolog (mouse) |
| SCP2 | sterol carrier protein 2 |
| PCBP1 | poly(rC) binding protein 1 |
| EIF1 | eukaryotic translation initiation factor 1 |
| SERPINI1 | serpin peptidase inhibitor, clade I (neuroserpin), member 1 |
| C3orf14 | chromosome 3 open reading frame 14 |
| ING1 | inhibitor of growth family, member 1 |
| MARC2 | mitochondrial amidoxime reducing component 2 |
| NMT2 | N-myristoyltransferase 2 |
| CTDSPL | CTD (carboxy-terminal domain, RNA polymerase II, polypeptide A) small phosphatase-like |
| AKAP5 | A kinase (PRKA) anchor protein 5 |
| SDF2L1 | stromal cell-derived factor 2-like 1 |
| RNF115 | ring finger protein 115 |
| B3GALT1 | UDP-Gal:betaGlcNAc beta 1,3-galactosyltransferase, polypeptide 1 |
| FAM86C1 | family with sequence similarity 86, member C1 |
| LRRC8D | leucine rich repeat containing 8 family, member D |
| DLX1 | distal-less homeobox 1 |
| GPBP1 | GC-rich promoter binding protein 1 |
| EIF4A2 | eukaryotic translation initiation factor 4A2 |
| IL1A | interleukin 1, alpha |
| SIRT1 | sirtuin 1 |

**Supplementary table 10.** miRNAs targeting SIRT1

| hsa-miR-34a-5p | hsa-miR-138-5p | hsa-miR-449b-5p |
| --- | --- | --- |
| hsa-miR-186-5p | hsa-miR-22-3p | hsa-miR-133b |
| hsa-miR-199a-5p | hsa-miR-181c-5p | hsa-miR-590-3p |
| hsa-miR-181a-5p | hsa-miR-519e-5p | hsa-miR-25-3p |
| hsa-miR-34c-5p | hsa-miR-518f-5p | hsa-miR-383-5p |
| hsa-miR-135a-5p | hsa-miR-373-3p | hsa-miR-486-5p |
| hsa-miR-92a-3p | hsa-miR-4262 | hsa-miR-204-5p |
| hsa-miR-543 | hsa-miR-155-5p | hsa-miR-199b-5p |
| hsa-miR-211-5p | hsa-miR-302a-3p | hsa-miR-363-3p |
| hsa-miR-7-5p | hsa-miR-449a |  |
